# Supplementary material for: Impact of l-Carnitine Supplementation on Liver Enzyme Normalization in Patients with Chronic Liver Disease: A Meta-Analysis of Randomized Trials
Source: J Pers Med. 2022 Jun 27;12(7):1053. doi: 10.3390/jpm12071053 (PMC9322040; doi:10.3390/jpm12071053)

## Slide 1
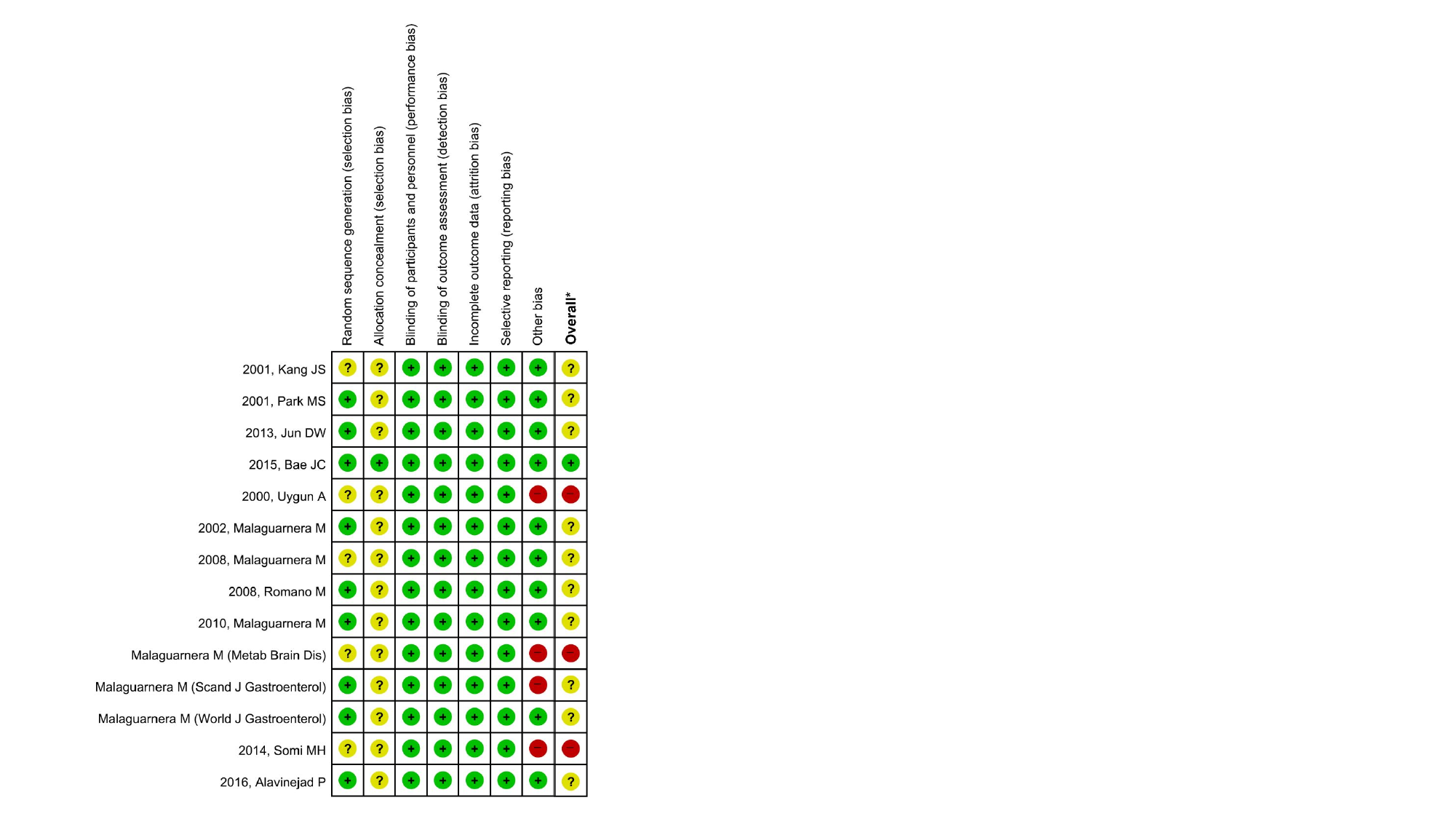

## Slide 2
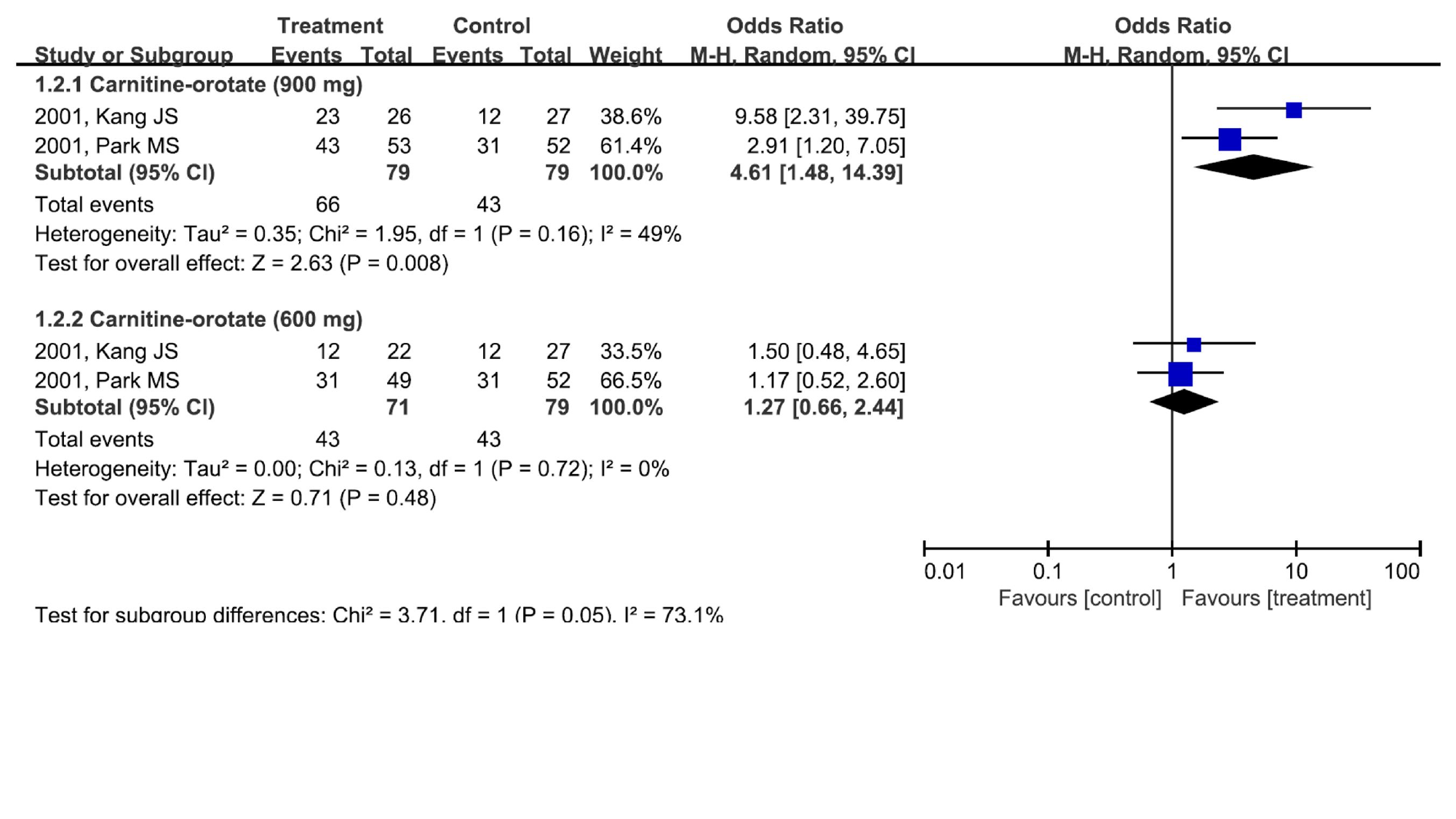

## Slide 3
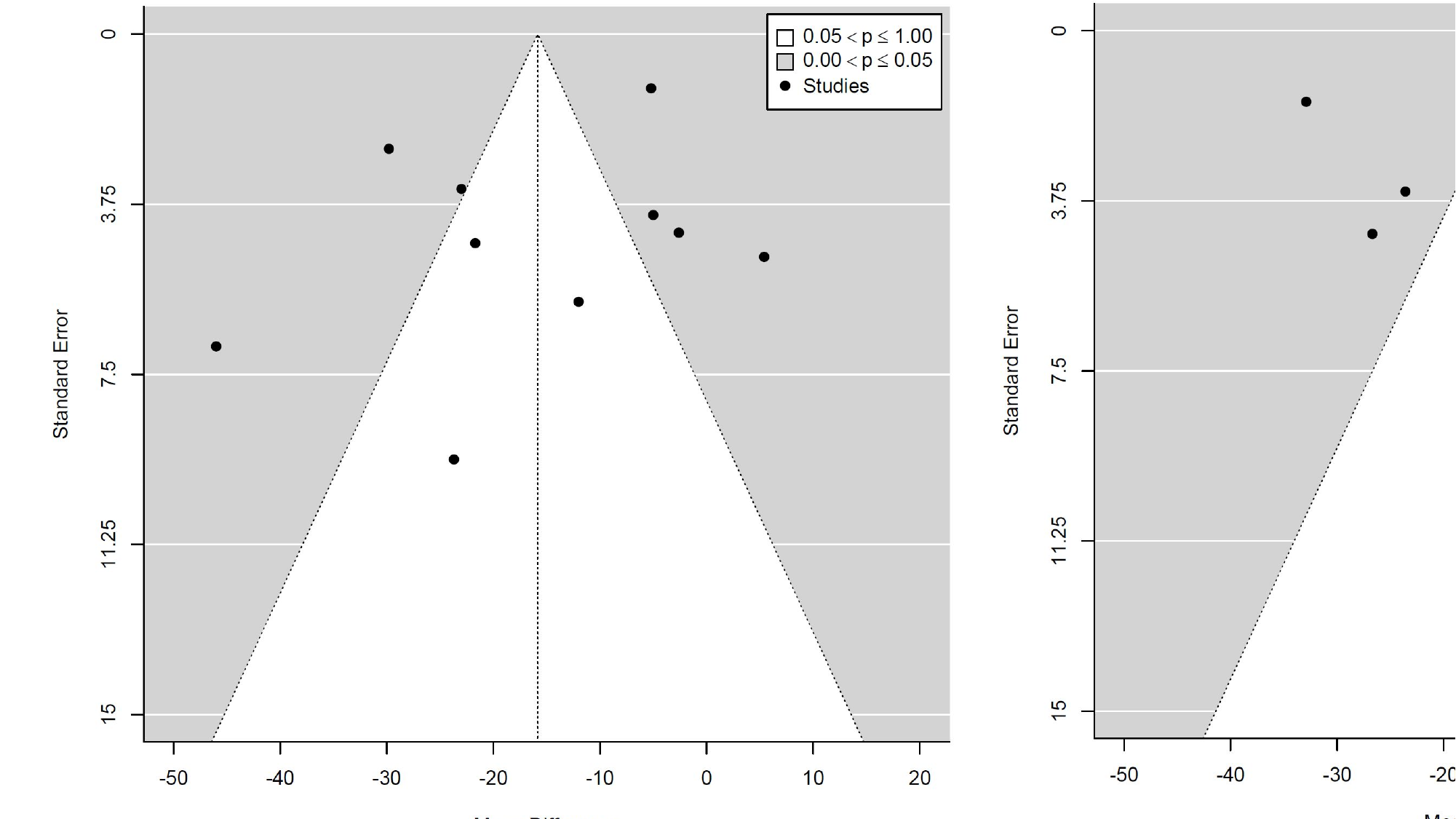

A
Post-treatment AST level
Post-treatment ALT level
B
Post-treatment AST level
Post-treatment ALT level

## Slide 4
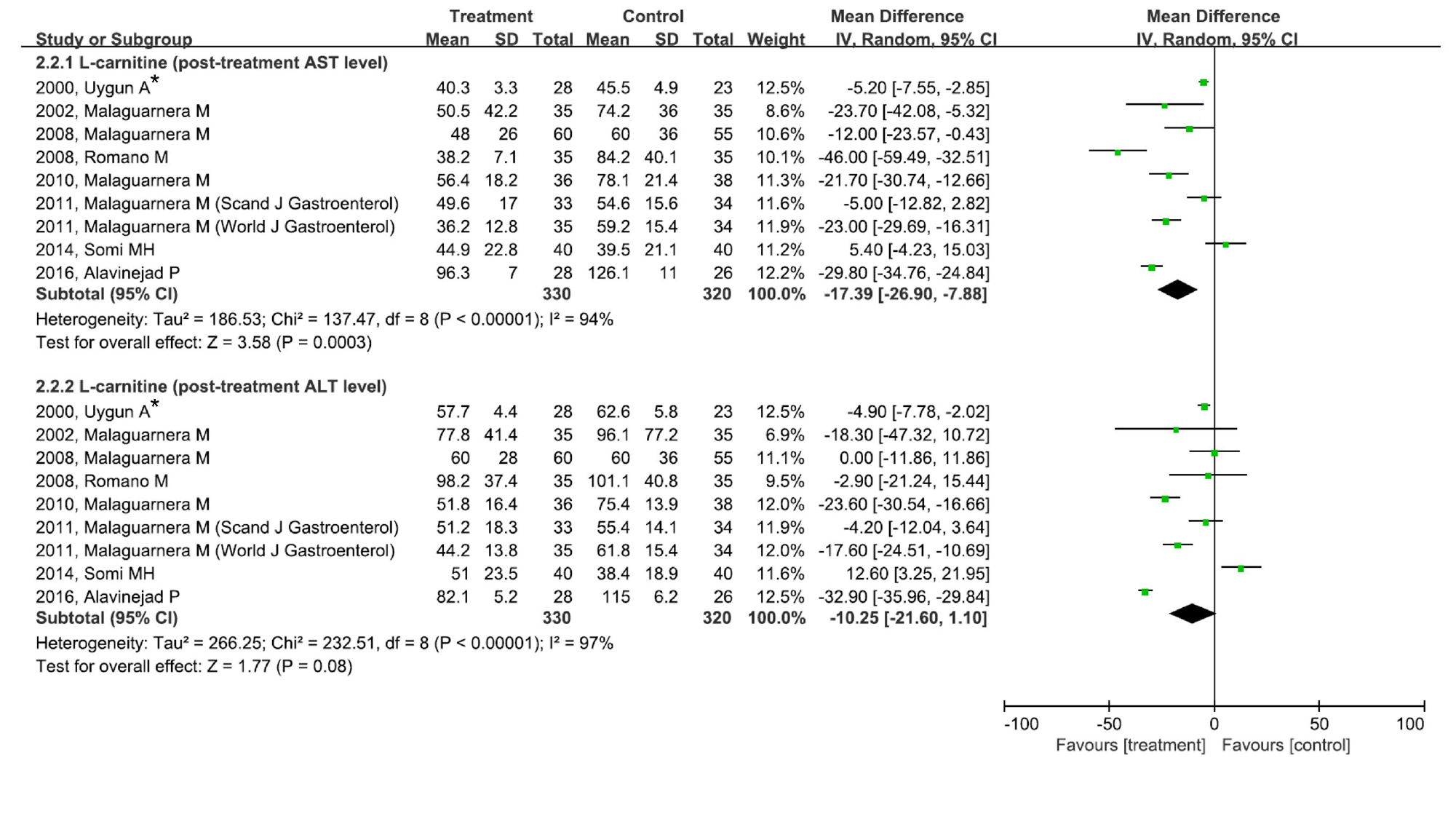

## Slide 5
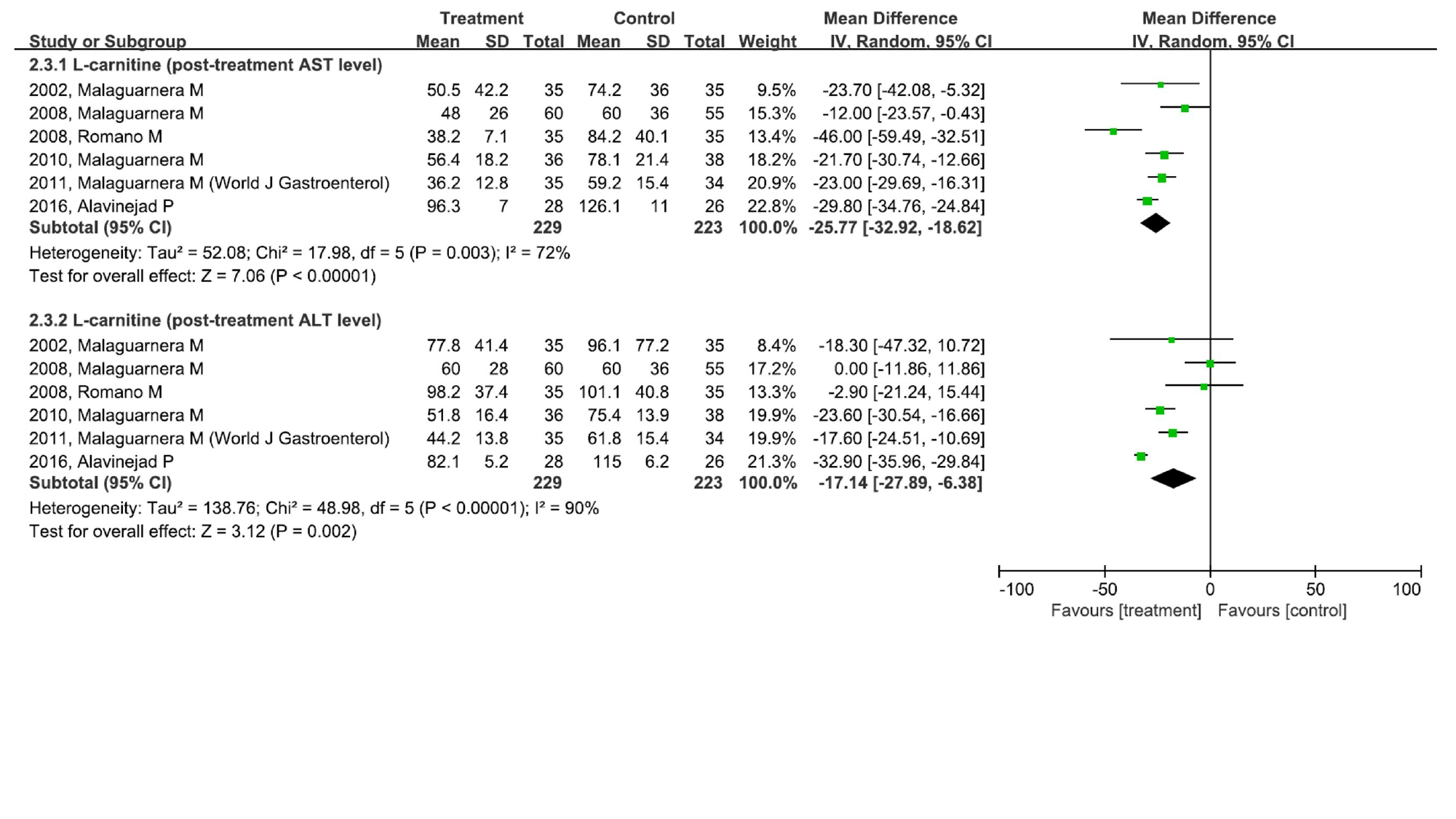

## Slide 6
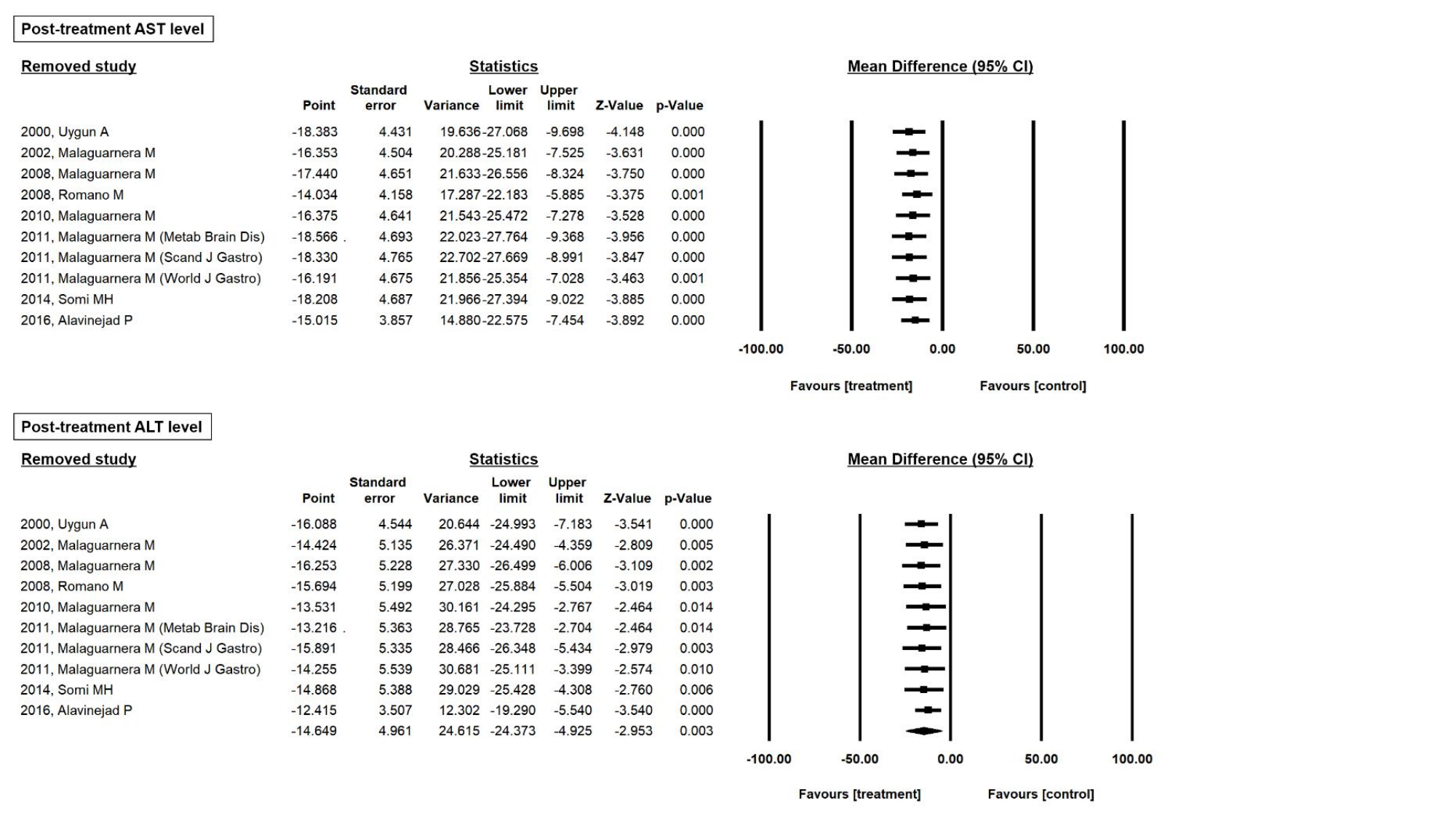

Supplement: Supplementary file 1 [file jpm-12-01053-s001.zip › jpm-1769710-supplementary.pptx]
